# An empirical examination of the conceptualization of companion animals

**DOI:** 10.1186/s40359-018-0228-1

**Published:** 2018-05-04

**Authors:** Ruben Hoffmann, Carl Johan Lagerkvist, Malin Hagberg Gustavsson, Bodil S. Holst

**Affiliations:** 1 Department of Economics, Swedish University of Agricultural Sciences, Box 7013, 75007 Uppsala, Sweden; 2 Department of Clinical Sciences, Swedish University of Agricultural Sciences, Box 7054, 75007 Uppsala, Sweden

**Keywords:** Attribute importance, Salience, Conceptualization, Cat, Dog, Companion animal

## Abstract

**Background:**

The extensive keeping of companion animals and the substantial monetary amount we spend on these animals indicate that they are highly valued. Although the benefits humans derive from keeping cats and dogs have been extensively studied, how we conceptualize these animals has received limited attention. How people conceptualize cats and dogs is important as it influences human behavior and the well-being of humans as well as animals. The objective of this paper was to examine the conceptual meaning of dogs and cats and the relative importance of meanings assigned to these species.

**Methods:**

Based on a Swedish on-line survey (*n* = 2028) the free-elicitation method was used to measure the salience of conceptualizations for dogs and cats as this method measures the accessibility of the focal object in people’s memory. An R-index approach was used to analyze the importance and dominance of attributes on the premise that the order in which attributes were listed by respondents reflects their relative importance. The sum of the choice probability was used to evaluate the stochastic rank order of attributes and Somers’ D was used to examine difference in rankings between groups of respondents.

**Results:**

For dogs, human well-being in terms of emotional and social support, and emotional attachment (*friendship*, *love*, *companionship*, *joy* and *loyalty*) were found to be most important while elements related to the animals themselves (e.g. *personality* of the animal) were found to be less important. For cats, *personality* of the animal was along with *love* found to be most important. The results were largely consistent across different types of households.

**Conclusions:**

The results provide information on the relative importance of salient attributes and thus indicate which attributes that are important to consider, for example, when analyzing human-animal interaction, animal welfare, human health and subjective-well-being, or the economic value of cats and dogs.

**Electronic supplementary material:**

The online version of this article (10.1186/s40359-018-0228-1) contains supplementary material, which is available to authorized users.

## Background

Cats and dogs are extensively kept as companion and working animals around the world. In Sweden and in Europe approximately one fourth [1, 2] and in the U.S. about one third of the households kept a cat or a dog [3] in 2012. In Europe, approximately € 35 billion was in 2016 spent on food products for companion animals and pet-related products and services [1]. Corresponding figure for the U.S. was approximately $ 67 billion [4].

The benefits humans derive from keeping cats and dogs have been extensively studied. For example, cats and dogs have been found to promote psychological health and well-being by providing companionship, emotional and social support, a sense of safety and security, entertainment, happiness, and relaxation (see e.g., [5–7]). Dogs have also been found to promote exercise and outdoor activities, affect the physical health of humans, and are used in different types of therapeutic settings (see e.g., [8, 5]). However, the scientific support for several of these benefits is disputed [9–11].

With different familiarity, and on the range from a specific animal to animals in general, people make associations between the identity of a focal object (e.g. a specific animal) and other conceptual associations held in mind. For example, a certain dog breed might be thought of as ‘caring’ or ‘cute’; another dog breed can be associated with being ‘aggressive’. These types of associations might be obtained as social constructs or successively learnt from internal experiences. At some point, the identity of the object and the associated conceptualizations become aligned in the mind of us as individuals. When considered in relation to a certain focal object the conceptualization establishes bearers of assigned meaning which, in turn, can be decomposed into three dimensions: functional; emotional; and abstract [12, 13]. In this way, this meaning may influence human behavior and well-being in relation to the focal object, and may therefore have the potential to ultimately affect how animals are treated, selected and cared for. Furthermore, not all bearers of assigned meaning are of equal importance.

Previous studies have examined the reasons people keep companion animals [14, 15], the different roles companion animals play in peoples’ lives [16, 17], the dimensions underlying the dog-human relationship [18], the types of economic values they provide [19], the consumption opportunities that they provide [20], and consumption values [21]. Previous studies have mainly focused on animal owners, most of them are qualitative in nature, but to our knowledge there are no previous studies on the conceptualization of cats and dogs.

The objective of this paper was to examine the conceptual meaning of dogs and cats, to explore how such meaning can be decomposed into abstract, emotional, and functional dimensions, and to examine the order of importance given to the identified elements of conceptual meaning.

## Methods

The free-elicitation method was used to measure the salience of conceptualizations for dogs and cats because it measures the accessibility of the focal object in people’s memory [22, 23]. In this respect, the concept of salience refers to the ease to which certain aspects (henceforth: attributes) of the focal object come to mind when thinking about the object. The free-elicitation method uses open-ended questions to let individuals indicate which features of an object are considered important [24]. Although importance can be influenced by different factors (e.g. distinctiveness) it is in this method assumed that the order of elicitation reflects importance, i.e. the top-of-mind features are the most important [25]. Salient attributes are more important than non-salient attributes [24, 26], i.e. attributes that come to mind are more important than those that do not and all non-salient attributes are equally (un)important.

### Questionnaire

An on-line questionnaire was designed to capture the attributes people associate with cats and dogs, respectively, as well as the relative importance of these attributes, and characteristics of respondents (see Additional file 1 for details on the questionnaire). Respondents answered questions either related to cats or to dogs and were, in an open-ended question, asked to list what came to mind when thinking of the species, describing each aspect in one or a few words. This question was phrased as openly as possible, to capture salience and minimize the problem of framing effects. Specifically, the question for dogs was: “Which aspects come to mind when you think of dogs? Describe separately each aspect in one word (or a few words)”. Respondents were initially provided two lines but for each response the respondent filled in, an additional line was provided. At most ten different responses could be listed. This structure was adopted to encourage respondents to only list the most salient aspects.

The dog questionnaire (cat questionnaire) included subsequent questions concerning whether the respondent kept a dog (cat), and if not if they had been thinking of doing so; which breeds they kept or had been thinking of keeping; reasons for keeping or not keeping a dog (cat); and if they had previously had a dog (cat) in the household. The last section of the questionnaire concerned socio-demographic information including age and gender of the respondent, size of the household, and whether the household included children.

### Data collection

Data consisted of a convenience sample of the Swedish population. Data was collected via an online questionnaire between the 10th of July and the 29th of September 2014. A link to the questionnaire was distributed via Facebook. No specific group of society was targeted. Although some self-selection bias was expected, in terms of a higher response rate among those positive towards dogs and cats, the data was expected to reflect many of the attributes that Swedes associate with cats and dogs. Respondents answered anonymously. The final sample, excluding respondents younger than 20 years old, consisted of 1267 respondents answering the dog questionnaire and 760 respondents answering the cat questionnaire. Descriptive statistics of the samples are presented in Table 1.

**Table 1:** Descriptive characteristics of the samples, percentages

		Dog questionnaire		Cat questionnaire
		(*n* = 1267)		(*n* = 760)
Number of animals in household				
0	…dogs	20	…cats	23
≥ 1	…dog	80	…cat	77
1	…dogs	41	…cats	32
2	…dogs	23	…cats	23
≥ 3	…dogs	13	…cats	18
N/A		3		4
Both cat and dog in household		31		13
Do not keep but has				
…previously kept a	…a dog^a^	71	… a cat^a^	83
…have thought of keeping	…a dog^a^	70	… a cat^a^	66
Age, years				
20–29		17		14
30–39		12		10
40–49		16		18
50–65		37		38
> 65		11		12
N/A		7		8
Gender				
Female		86		86
Male		7		6
N/A		7		8
Persons in household				
1		17		21
2		41		39
3–4		27		26
> 4		8		6
N/A		7		8
Children in household				
Yes		31		30
No		62		62
N/A		7		8

Percentage in proportion of total sample except otherwise noted, ^a^are proportion of respondents not keeping a dog (or cat) who answered whether or not previously kept and whether or not have been thinking of keeping, respectively

A large proportion, roughly four out of five, of the respondents of the dog (cat) questionnaire kept a dog (cat). Of those that did not, a majority had previously kept or had been thinking of keeping a dog (cat). Official statistics on the socio-demographic characteristics of the Swedish population that keep cats and dogs do not exist. Hence, the characteristics of the sample were compared to the distribution of the Swedish population as a whole [27]. The age groups 30–39 and older than 65 were underrepresented while the age group 50–65 was overrepresented. Women were overrepresented as were smaller household sizes (especially two person households). The proportion of households with children reflected the proportion in Sweden [28].

### Categorization, classification and ranking of responses

The key question of the questionnaire was the open-ended question concerning what came to mind when thinking of dogs or cats. Responses were transformed and categorized for data analysis. First, terms with similar semantic meaning (e.g. “friendly”, “kind”, and “warm-hearted”) were grouped together. Then groups of words interpreted as having the same association and connotation (conceptual meaning) were categorized under a common heading (e.g., “stubborn”, “strong willed”, “proud”, and “integrity” were grouped under personality/mentality of the animal). When a response could be interpreted as belonging to two categories it was included in both categories. When terms with similar association and connotation (conceptual meaning) were listed separately by a large proportion of respondents, these terms were categorized separately. Finally, each category was classified as functional, emotional, or abstract according to the value dimension [14].

The attributes were ranked in the order in which they were listed. Given the large number and heterogeneity of the responses, it was inevitable that some respondents mentioned more than one attribute within a specific category. In these cases, the response with the highest rank was used in the statistical analysis. Respondents could list up to 10 attributes. Hence, attributes not mentioned by a respondent were ranked as 11 in order to account for that attributes that do not come to mind are equally unimportant and less important than attributes that do come to mind.

### Data analysis

An R-index [28] approach was used to analyze the importance and dominance of attributes on the premise that the order in which attributes were listed by respondents reflects their relative importance. The R–index expresses the pairwise probability that a given attribute is preferred over another.

Aggregating the rank of each attribute over all respondents results in a response matrix as shown in Table 2, with *S*_*m*_ being the sum of counts of all ranks of each attribute *m* = 1, 2, …, *M* (see e.g., [29]).

**Table 2:** Response matrix used for computing the R-index

	Counts ranked as					
	1st	2nd	…	10th	11th (not mentioned)	Sum
Attribute 1 (A_1_)	a_1_	b_1_	…	j_1_	k_1_	S_1_
Attribute 2 (A_2_)	a_2_	b_2_	…	j_2_	k_2_	S_2_
…	…	…	…	…	…	…
Attribute 30 (A_30_)	a_30_	b_30_	…	j_30_	k_30_	S_30_

Let the pairwise probability that attribute *m* is perceived as more important than attribute *n* be given by *R*_*mn*_, with *m*, *n* = 1, 2, …, *M*. The probability that attribute 1 is preferred over attribute 2 is given by $\frac{A}{S_1{S}_2}$ with *A* = *a*_1_(*b*_2_ + *c*_2_ + … + *k*_2_) + *b*_1_(*c*_2_ + … + *k*_2_) + … + *j*_1_*k*_2_ while the probability of attribute 2 being preferred over attribute 1 is given by $\frac{C}{S_1{S}_2}$ with *C* = *a*_2_(*b*_1_ + *c*_1_ + … + *k*_1_) + *b*_2_(*c*_1_ + … + *k*_1_) + … + *j*_2_*k*_1_. Furthermore, the probability that attribute 1 is equally preferred to attribute 2 is given by $\frac{B}{S_1{S}_2}$ with *B* = (*a*_1_*a*_2_ + *b*_1_*b*_2_ + … + *k*_1_*k*_2_). The pooled probability that attribute 1 is more important than attribute 2 is then given by ${R}_{12}=\frac{A+0.5B}{2}$ [29, 30].

The pairwise probability *R*_*mn*_ reflects the relative importance of an attribute and Table 3 shows the (m*m) matrix of the R-index values for all pairwise comparisons of attributes, with *R*_*mm*_ = 100. Based on the R-index values, two-tailed pairwise tests of whether attribute *m* was preferred over attribute *n* or whether attribute *n* was preferred over attribute *m*, were performed using the critical values derived by Bi and O’Mahony [31]. The null hypothesis that the R-index equals the chance value of 50% is rejected if the R-index is significantly greater than the chance value (H_0_: *R*_*mn*_ = 50). An attribute *m* is considered strictly dominant if it is preferred over all other attributes, i.e. *R*_*mn*_ – 50 > critical value for all other *n*≠*m* attributes. An attribute *m* is considered weakly dominant if it dominates at least one other attribute (i.e. *R*_*mn*_ – 50 > critical value for at least one attribute *n*≠*m*), while not being dominated by any other attribute (i.e. – *R*_*nm*_ – 50 < critical value is not the case for any *n*≠*m* attributes).

**Table 3:** R-index matrix with choice probabilities

Attribute A_m_	A_1_	A_2_	…	A_M-1_	A_M_	Sum of choice probability (SCP)
A_1_	100	R_12_	…	R_1(M-1)_	R_1M_	SCP_1_ = 100 + R_12_ + R_13_ + … + R_1M_
A_2_	R_21_	100	…	R_2(M-1)_	R_2M_	SCP_2_ = R_21_ + 100 + R_23_+ … + R_2M_
…	…	…	…	…	…	…
A_M-1_	R_(M-1)1_	R_(M-1)2_	…	100	R_(M-1)M_	SCP_M_ = R_M1_ + … + 100+ R_(M-1)M_
A_M_	R_M1_	R_M2_	…	R_M(M-1)_	100	SCP_M_ = R_M1_+ … + R_M(M-1)_ + 100

The sum of the choice probability (SCP) that attribute *m* was more important than attribute *n* was obtained by summing the pooled pairwise probabilities by row as shown in Table 3. The SCPs can be used to evaluate the stochastic rank order of attributes [30]. They also enable pairwise comparisons of the relative importance of two attributes that goes beyond the rankings.

Rankings were obtained for different groups of respondents. Differences between subgroups were examined for specific attributes based on Somers’ D [32]. Taking potential ties in ranking into account Somers’ D was used to measure the association between a group variable and the ranking of an attribute A_m_. It provides information on the difference between the probability that a randomly selected person from subgroup *X* rank attribute A_m_ higher than a randomly selected person from subgroup *Y* and the probability that a person in subgroup *X* rank A_m_ lower than a person in subgroup *Y*. Hypotheses of no differences between subgroups were, for each of the top attributes, tested based on Somers’ D (1% level of significance) and 99% confidence intervals were presented for cases with statistically significant differences.

## Results

### Categorization and classification of responses

Of the respondents of the dog questionnaires, 50% listed one or two attributes, 37% three or four attributes while 14% listed five or more attributes. Corresponding figures for the cat questionnaire were 59%, 34%, and 7%. Following the procedure described in section 2.3, the responses for dogs were sorted into 25 attribute categories. The same categories were included for cats with the exceptions of *physical activity*, *outdoor activity* and *leisure activities* as responses in these categories were lacking. The conceptual dimensions (functional, emotional or abstract) and the percentage of responses in each of the attribute categories are presented in Table 4.

**Table 4:** Attribute categories with value dimension and percentage of respondents mentioning each attribute

	Attribute category	Value dimension^a^	% of respondents in	
			dog questionnaire	cat questionnaire
1	Companionship	F	31%	18%
2	Family	F	7%	2%
3	Friend	F	35%	4%
4	Social interaction	F	7%	1%
5	Relaxation	F	2%	6%
6	Human health	F	4%	4%
7	Physical activity	F	14%	0%
8	Outdoor activities	F	7%	0%
9	Leisure activities	F	11%	0%
10	Services provided as working animals etc.	F	6%	3%
11	Responsibility	F	11%	5%
12	Demanding	F	6%	3%
13	Nuisances	F	5%	8%
14	Safe/unsafe	E, F	9%	2%
15	Central in life	E, F	5%	2%
16	Love	E	35%	51%
17	Joy	E	31%	11%
18	Enjoyable	E	7%	14%
19	Loyalty	A, E	24%	3%
20	Honesty	A, E	3%	2%
21	Appearance	A, E	3%	18%
22	Personality (including mentality)	A, E	3%	56%
23	Intelligence	A	4%	10%
24	Breeds & other traits	A	5%	9%
25	Animal welfare	A	2%	4%

^a^F refers to the functional, E refers to the emotional and A refers to the abstract value dimension

A majority of the attributes were classified as having a functional value dimension although attributes with emotional and abstract value dimensions were also found. The latter primarily concerned animal traits without a clear functional or emotional value dimension. It should be noted that the value dimensions are not distinctly separate. Some attributes had more than one potential value dimension. For example, watch dogs were classified as functional (guard the house) as well as emotional (providing a feeling of being safe).

### The relative importance of dog attributes

**Fig. 1:**
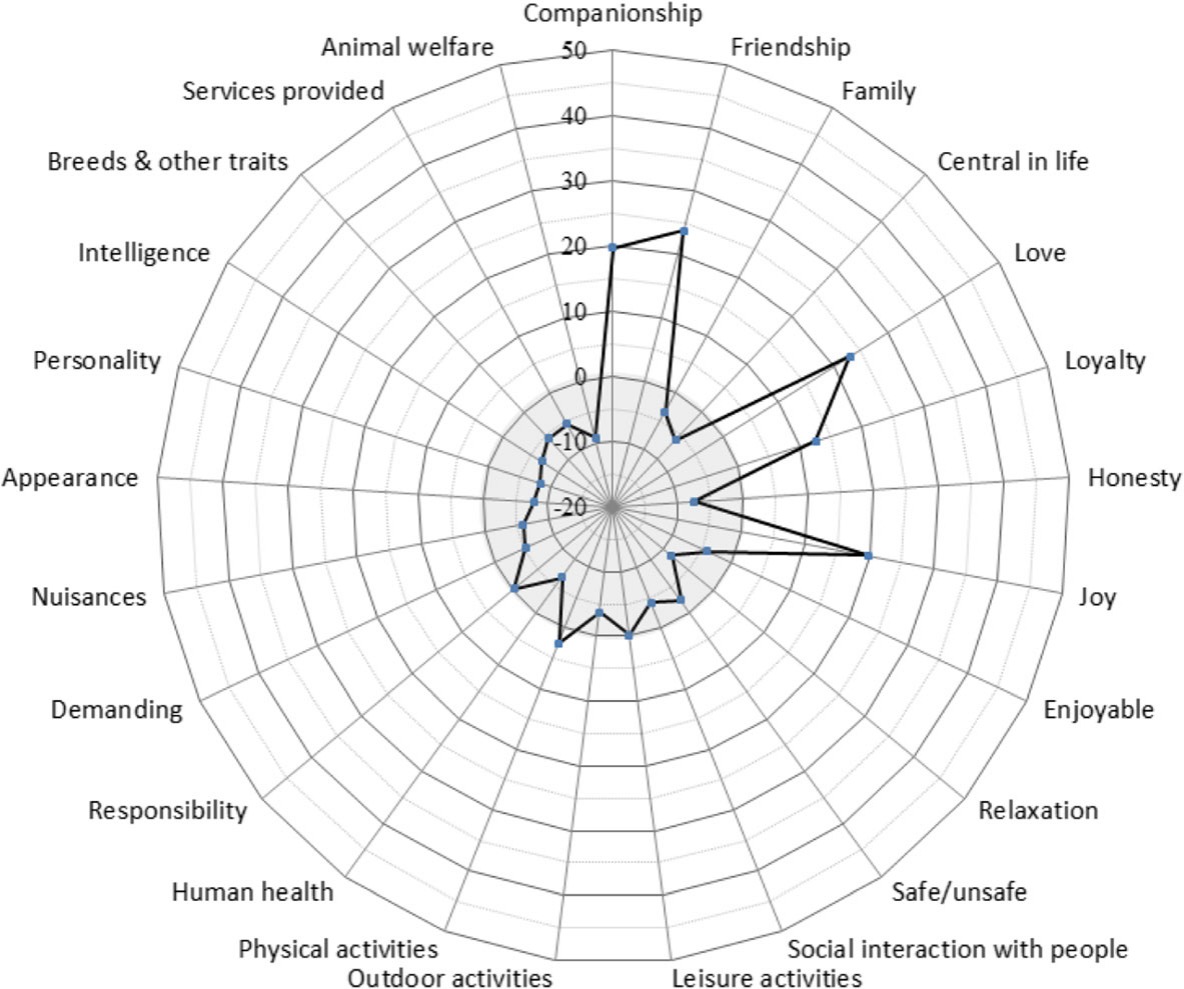
Importance of dog attributes relative to chance values. The chance value refers to the situation where each of the attributes has a 50–50 chance of being preferred over each of the other attributes. For (positive) values outside the grey circle the attribute has a probability greater than 50% to be considered important and for (negative) values inside the circle a probability less than 50%

The sum choice probabilities and the rankings of dog attributes are presented in Table 5. Based on the obtained sum choice probabilities Fig. 1 depicts the importance of each attribute relative to the chance value, i.e. a situation where each attribute has a 50–50 chance of being preferred over each of the other attributes.

**Table 5:** Rank, choice probabilities and dominance based on R-index for all respondents

Rank	Dogs (*n* = 1267)	Dominance				Cats (*n* = 760)	Dominance			
	Attributes	SCP	AD	DA		Attributes	SCP	AD	DA	
1	Friendship	1608	21	0	^a^	Personality (incl. mentality)	1667	21	0	^b^
2	Love	1599	21	0	^a^	Love	1600	20	1	
3	Companionship	1557	21	0	^a^	Companionship	1231	17	2	
4	Joy	1557	21	0	^a^	Appearance	1224	17	2	
5	Loyalty	1465	20	4		Enjoyable	1185	14	2	
6	Physical activities	1333	17	5		Joy	1147	11	4	
7	Leisure activities	1298	12	5		Intelligence	1135	8	4	
8	Responsibility	1295	12	5		Breeds & other traits	1124	5	4	
9	Safe/unsafe	1271	6	6		Nuisances	1121	5	5	
10	Family	1254	2	6		Relaxation	1094	0	5	
11	Outdoor activities	1252	2	6		Responsibility	1087	0	5	
12	Enjoyable	1248	1	6		Friendship	1078	0	6	
13	Social interaction	1245	1	6		Health	1076	0	6	
14	Demanding	1233	0	8		Animal welfare	1073	0	6	
15	Services provided	1228	0	8		Services provided	1068	0	7	
16	Breeds & other traits	1227	0	8		Demanding	1066	0	7	
17	Central in life	1222	0	8		Loyalty	1064	0	7	
18	Nuisances	1222	0	8		Safe/unsafe	1056	0	9	
19	Health	1213	0	8		Honesty	1055	0	9	
20	Intelligence	1208	0	9		Central in life	1050	0	9	
21	Honesty	1201	0	9		Family	1050	0	9	
22	Appearance	1199	0	9		Social interaction	1047	0	9	
23	Personality (incl. mentality)	1193	0	9						
24	Relaxation	1190	0	11						
25	Animal welfare	1181	0	13						

*Rank* rank according to the choice probability, *SCP* sum choice probability, *AD* number of other attributes dominating, *DA* number of other attributes dominated by, ^a^denotes weak dominance, and ^b^denotes strict dominance

As shown in Table 5, the five attributes ranked highest according to the sum choice probabilities were *friendship*, *love*, *companionship*, *joy* and *loyalty*. These dominated at least 20 other attributes and the first four attributes showed weak dominance. About half of the attributes did not dominate any other attribute. *Animal welfare* and several animal traits were among the less important attributes. The top ranked attribute *friendship* was 36% more likely than the least important attribute (*animal welfare*) to be considered important while the attributes *love* (35%), *companionship* (32%), *friendship* (32%), and *loyalty* (24%) were more than 20% more likely to be considered important. Attributes related to the abstract value dimension (e.g. animal welfare and characteristics of the animal) were generally ranked low while the highest ranked attributes primarily had functional and/or emotional value dimensions.

Subgroups of respondents were formed to examine if the attributes which individuals found important were influenced by the characteristics of the household, the characteristics of the respondent, whether the respondent had a dog or not, and if they did not, whether they had been thinking of keeping a dog or not. The sum choice probabilities, the rankings and the dominance of the attributes in each of the subgroups are presented in Table 6.

**Table 6:** Dogs: Rank, choice probabilities and dominance based on R-index for different subgroups

	Keeping a dog						Gender				Household size				Children in household			
	Yes (*n* = 1016)		No but have thought of (*n* = 153)		No & have not thought of (*n* = 65)		Female (*n* = 1086)		Male (*n* = 90)		Single (*n* = 221)		Not single (*n* = 955)		Yes (*n* = 393)		No (*n* = 783)	
Rank	Attr	SCP	Attr	SCP	Attr	SCP	Attr	SCP	Attr	SCP	Attr	SCP	Attr	SCP	Attr	SCP	Attr	SCP
1	3^a^	1618	17^a^	1596	3^a^	1548	3^a^	1619	19^a^	1575	1^a^	1658	16^a^	1610	16^a^	1651	3^a^	1608
2	16^a^	1613	3^a^	1593	16^a^	1520	16^a^	1613	1^a^	1574	3^a^	1638	3^a^	1607	3^a^	1622	16^a^	1581
3	1^a^	1560	1^a^	1571	1^a^	1474	17^a^	1574	3^a^	1549	16^a^	1577	17^a^	1567	1^a^	1569	17^a^	1568
4	17	1560	16^a^	1562	19^a^	1469	1^a^	1566	16^a^	1496	17^a^	1537	1^a^	1545	17^a^	1548	1^a^	1565
5	19	1460	19^a^	1519	13^a^	1424	19	1457	17^a^	1412	19	1422	19	1476	19	1513	19	1443
6	7	1336	14	1334	17	1367	7	1341	14	1342	7	1361	7	1328	9	1327	7	1340
7	9	1313	7	1305	14	1356	11	1300	9	1308	9	1321	11	1307	7	1324	11	1301
8	11	1298	12	1288	7	1331	9	1296	10	1297	4	1288	9	1292	14	1308	9	1283
9	2	1259	11	1275	4	1312	14	1263	11	1283	8	1265	14	1277	11	1295	18	1255
10	8	1258	18	1269	11	1311	8	1255	4	1272	2	1262	2	1252	8	1254	2	1253
11	14	1252	23	1266	24	1293	2	1251	2	1270	18	1258	8	1249	2	1253	8	1252
12	4	1247	2	1258	20	1274	18	1245	24	1269	11	1257	18	1238	4	1240	14	1250
13	18	1245	13	1245	23	1236	4	1242	13	1267	10	1241	4	1237	13	1236	4	1247
14	15	1230	10	1241	22	1234	12	1233	23	1260	6	1236	12	1234	12	1227	12	1236
15	10	1226	9	1240	9	1233	15	1222	7	1257	14	1235	24	1228	10	1217	24	1233
16	12	1224	24	1233	21	1233	24	1222	12	1227	12	1216	15	1228	18	1216	10	1232
17	24	1224	21	1227	12	1232	10	1221	8	1216	24	1216	13	1227	15	1212	6	1226
18	6	1223	8	1224	25	1218	13	1218	5	1215	20	1200	10	1224	24	1211	15	1226
19	13	1206	4	1192	10	1217	6	1213	6	1215	23	1198	23	1209	20	1195	13	1214
20	20	1199	20	1191	15	1217	20	1201	15	1213	13	1197	6	1205	23	1189	23	1211
21	23	1196	22	1186	18	1217	23	1199	18	1203	15	1192	20	1200	6	1188	21	1206
22	5	1193	15	1177	5	1216	21	1194	25	1203	21	1191	21	1196	5	1182	20	1202
23	21	1192	25	1175	2	1196	22	1190	21	1201	22	1189	5	1193	25	1177	22	1197
24	22	1191	6	1170	8	1195	5	1186	20	1189	25	1179	22	1190	22	1174	5	1192
25	25	1180	5	1161	6	1178	25	1178	22	1189	5	1168	25	1181	21	1173	25	1182

*Rank* denotes rank according to the SCP, *Attr* denotes the attribute as numbered in Table 4, *SCP* denotes sum choice probability, ^a^denotes weak dominance

The same top five attributes were found regardless of whether respondents were keeping a dog or not and irrespective of household size, gender, and whether the household included children or not. However, the results suggest some differences between the subgroups in the relative importance of specific attributes. In the following we focus on attributes with a probability greater than the chance value.

Fewer dominant attributes could be observed for people having a dog (*friendship, love, companionship)* than for people not having but that had been thinking of keeping a dog (*joy, friendship, companionship, love, loyalty*) or that had not been thinking of keeping a dog (*friendship, love, companionship, loyalty, nuisances*). The results indicated that the most important attributes for people keeping a dog were important also for people that neither had kept nor had been thinking of keeping a dog, although to a lesser extent (with the exception of *loyalty*). Furthermore, a larger relative importance of the attribute *nuisances* could be observed in the latter group and the attribute *safe/unsafe* was more emphasized among people not having a dog than for people having dogs. Statistical tests based on Somers’ D suggest that, given a randomly selected person who has a dog and a randomly selected person who does not have and has not been thinking of getting a dog, the former is 16%, 99% CI [3%, 28%], more likely to rank *joy* higher than vice versa while the latter is 16%, 99% CI [3%, 28%], more likely to rank *nuisances* higher than vice versa.

The results also indicate that the attributes *loyalty* and *safe/unsafe* were more important for males than females while the opposite is the case for the attributes *love, joy, friendship* and, *physical activities*. Statistical tests suggest that, given a randomly selected woman and a randomly selected man, a woman is 14%, 99% CI [3%, 25%], more likely to rank *joy* higher than vice versa. Furthermore, the importance of the attribute *companionship* was more pronounced for single person households while the attributes *love* and *loyalty* were more pronounced for households with children.

### The relative importance of cat attributes

**Fig. 2:**
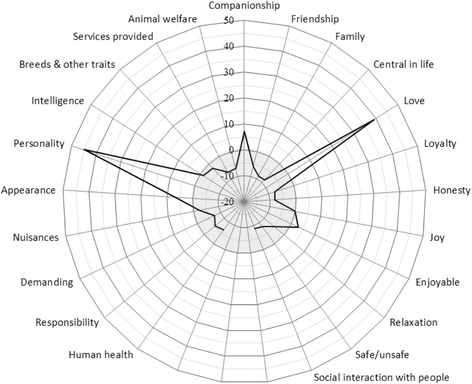
Importance of cat attributes relative to chance values. The chance value refers to the situation where each of the attributes has a 50–50 chance of being preferred over each of the other attributes. For (positive) values outside the grey circle the attribute has a probability greater than 50% to be considered important and for (negative) values inside the circle a probability less than 50%

The sum choice probabilities, rankings and dominance of cat attributes are presented in Table 5 and the importance of each attribute relative to the chance value, i.e. a situation where each attribute has a 50–50 chance of being preferred over each of the other attributes, is presented in Fig. 2.

The two top ranked attributes were *personality* (including mentality) of the animal and *love*. These attributes were considerably more important than any of the other attributes. *Personality* was 59% more and *love* was 53% more likely to be considered important than the least important attribute (*social interaction*), and both top attributes were at least 30% more likely to be considered important than the attribute ranked third (*companionship*). The attribute *personality* strictly dominated all other attributes and the attribute *love* dominated all other attributes except the top ranked. A majority of the attributes did not dominate any other attribute. The two most important attributes had both abstract and emotional value dimensions but not a clear functional dimension. Examples of less important attributes were *social interaction*, *family, friendship*, *animal welfare*, and *loyalty*.

The sum choice probabilities, rankings and dominance of attributes in different subgroups are presented in Table 7. *Personality* of the animal and *love* were the highest ranked attributes regardless of whether respondents were keeping a dog or not and irrespective of household size, gender, and whether the household included children or not. Dominance was in all of the subgroups detected for one or both of these attributes. Attributes that were less important in all subgroups included *family, friendship, social interaction,* and *loyalty*. As for dogs, the results do however suggest some differences in the relative importance of specific attributes between the subgroups.

**Table 7:** Cats: Rank, choice probabilities and dominance based on R-index for different subgroups

	Keeping a cat						Gender				Household size				Children in household			
	Yes (*n* = 585)		No but have thought of (*n* = 99)		No & have not thought of (*n* = 51)		Female (*n* = 653)		Male (*n* = 46)		Single (*n* = 163)		Not single (*n* = 537)		Yes (*n* = 227)		No (*n* = 474)	
Rank	Attr	SCP	Attr	SCP	Attr	SCP	Attr	SCP	Attr	SCP	Attr	SCP	Attr	SCP	Attr	SCP	Attr	SCP
1	22^b^	1670	22^a^	1766	22^a^	1519	22^b^	1688	22^a^	1659	22^a^	1619	22^b^	1705	22^b^	1690	22^b^	1681
2	16	1597	16^a^	1717	16^a^	1446	16	1613	16^a^	1438	16^a^	1581	16	1608	16	1567	16	1619
3	21	1227	1	1210	1^a^	1299	1	1225	1	1236	21	1244	1	1227	21	1251	1	1233
4	1	1226	21	1200	13	1285	21	1225	24	1235	1	1229	21	1220	1	1220	21	1213
5	18	1189	18	1163	21	1259	18	1182	21	1235	18	1210	18	1170	13	1176	18	1186
6	17	1151	24	1150	23	1231	17	1143	23	1190	17	1150	17	1139	18	1163	17	1150
7	23	1126	23	1138	18	1149	23	1128	13	1182	24	1136	23	1134	23	1142	23	1127
8	24	1124	17	1125	17	1126	24	1120	18	1143	23	1126	24	1125	24	1141	24	1121
9	13	1105	13	1116	6	1108	13	1111	12	1138	11	1104	13	1120	5	1126	11	1090
10	5	1097	5	1095	2	1106	5	1097	17	1136	13	1099	5	1102	17	1123	2	1089
11	11	1090	6	1084	11	1104	11	1092	10	1115	25	1093	11	1084	11	1086	13	1086
12	2	1083	12	1072	10	1103	2	1078	3	1109	6	1092	2	1075	10	1076	5	1083
13	25	1078	11	1070	24	1084	25	1077	5	1094	2	1085	6	1070	6	1075	25	1082
14	6	1072	20	1062	5	1084	6	1076	6	1068	5	1079	10	1068	12	1074	6	1075
15	10	1067	10	1062	4	1064	10	1066	2	1065	12	1073	25	1067	20	1057	19	1069
16	12	1066	25	1052	14	1064	19	1065	4	1046	10	1072	12	1064	4	1056	10	1067
17	19	1066	14	1052	19	1064	12	1061	11	1044	19	1060	19	1064	25	1055	12	1063
18	3	1056	19	1041	3	1041	20	1056	20	1043	3	1059	20	1056	2	1052	14	1061
19	14	1056	4	1039	12	1041	14	1056	14	1042	14	1059	14	1054	19	1052	15	1054
20	20	1054	2	1038	15	1041	15	1049	19	1042	20	1053	4	1052	14	1042	20	1054
21	15	1053	15	1029	20	1041	4	1047	15	1021	15	1045	15	1048	3	1042	3	1054
22	4	1047	3	1019	25	1041	3	1045	25	1021	4	1032	3	1048	15	1033	4	1043

*Rank* denotes the rank according to the SCP, *Attr* denotes the attribute as numbered in Table 4, *SCP* denotes sum choice probability, ^a^denotes weak dominance, and ^b^denotes strong dominance

Respondents that kept a cat or had been thinking of doing so emphasized the attributes *personality* of the animal and *love* more than persons who neither kept nor had been thinking of keeping a cat. Furthermore, the results indicate that *nuisances*, *companionship*, and *intelligence* were more important for persons who neither kept nor had been thinking of keeping a cat. Overall the relative importance of attributes was similar regardless of gender, whether or not in a single person household and whether or not there were children in the household. The results indicate that women emphasize the attribute *love* more than men, men emphasize the attribute *breed* more than women, single person households emphasize the attribute *personality* of the animal more than other households, and that *nuisances* are less important for households without children. However, no statistically significant (*P* < 0.01) difference between subgroups could, based on Somers’ D, be detected for the four top attributes.

## Discussion

How we conceptualize and perceive cats and dogs influences human behavior and emotions, and thus the well-being of both animals and humans. In this study the conceptualization of dogs and cats was empirically examined. Respondents were asked what comes to mind when thinking of dogs (cats) and the responses were sorted into 25 attribute categories which were categorized as having functional, emotional, or abstract dimensions. The relative importance of these attributes were then examined.

The results highlight the bearers of meanings assigned to cats and dogs. Dogs and cats have long lived close to humans, reflected in the present study through many of the expressions used to describe them, for example *companionship*, *love*, and *central in life*. The results suggest that attributes with an emotional value dimension are important for both species (for cats top two and for dogs top four) while there is a larger emphasis on the functional value dimension related to dog attributes and a larger emphasis on the abstract value dimension related to cat attributes. The important attributes with a functional dimension relate to recreational activities important for human well-being as do many attributes with an emotional dimension.

Although the animals satisfy many different human needs, this study suggests that a limited number of attributes are more appreciated, rather than the multitude of potential benefits suggested in literature. Roughly half of the respondents mentioned one or two attributes and more than 85% listed no more than four attributes. Furthermore, the statistical analysis revealed a limited number of dominant attributes, specifically 4–5 for dogs and 1–2 for cats. That dogs fulfill a more diverse set of human needs may partly explain this difference. For example, while many respondents mentioned physical, leisure or outdoor activities in relation to dogs, none did in relation to cats. Another indication that dogs fulfill a more diverse set of human needs than do cats is a more gradual decline in the relative importance of dog attributes.

For dogs the most important attributes were *companionship*, *friendship*, *love*, *joy*, and *loyalty*. For cats the most important attributes were *personality* of the animal and *love*. Given the explorative approach adopted in this study it was not possible to conduct direct statistical comparisons between cats and dogs. Nevertheless, it is interesting to note that the results indicate that *love* was by all types of respondents ranked among the most important attributes for both cats (top two) and dogs (top four). The strong emotional connection has implications for the well-being of animals and plays a role in psychological processes that affect veterinary medicine [33]. The connection may help explain the increased spending on veterinary services related to these species [34]. Another possible consequence of a strong emotional connection is humanization of animals. In combination with owners’ lack of knowledge of animal behavior and training humanization may lead to negative outcomes such as behavioral abnormalities. These may in turn result in physical, emotional and economic costs [35]. Animals exhibiting e.g. aggressive behavior, counteracting highly ranked attributes, are often relinquished. In England, behavioral abnormalities is the most common cause of death in dogs less than 3 years old attending primary veterinary practices [36], and it is the most common cause for relinquishment to shelters in the US [37].

It is also noteworthy that the most important cat attribute (*personality* of the animal) was among the least important dog attributes. The latter suggests that the *personality* of dogs is not highly valued as an attribute per se, although it indirectly influence many other attributes valued by humans. Possibly, evolution, as well as reasons for domestication and breeding, contribute to the differences in how we perceive cats and dogs, and may also influence our expectations. Cats are solitary animals, while dogs live in social groups. Dogs have been selected for functions that to a large extent are performed in relation to humans (reflected in e.g. companionship, friendship and loyalty). Cats, on the other hand, perform their function as pest controllers independently, and are associated with attributes such as integrity.

The ranking of attributes showed a high degree of similarity between different types of individuals. Interestingly, the attributes important for people that kept a dog (cat) were also important for people not having a dog (cat), even people who had not been thinking of keeping a dog (cat). This suggests that there are potentially substantial positive externalities related to non-owners which are important to take into account when analyzing for example how humans perceive and value dogs and cats as it has implications on the well-being of humans as well as animals. Less surprising was that people who did not keep a dog (cat), especially those that had not been thinking of keeping a dog (cat), emphasized *nuisances* more.

Another reflection is that *animal welfare* as a specific attribute was ranked among the least important attributes for both dogs and for cats. This does however not mean that the well-being of cats and dogs are not perceived as important. The relative importance of attributes such as *friendship* and *love* signal strong emotional connections and as previously mention this has implications for the well-being of animals.

Regarding dogs, *companionship* was more important for single person households while *love* and *loyalty* were more important for households with than without children. Furthermore, men emphasized *loyalty* and *safety* more than women while *love, joy, friendship* and, *physical activities* were more important for women than for men. The differences between genders may partly be explained by respondents potentially thinking of different breeds or differences in expectations e.g. taking a more pragmatic, functional approach to dog ownership. For cats it can be noted that women emphasized the attribute *love* more than men while men emphasized the attribute *breed* more than women.

A majority of the attributes did not dominate any other attribute which can be expected given the large proportion of ties in the data set. This finding relates to the use of the open-ended format. The alternative of providing pre-specified lists are more prone to response biases in form of yea-saying (i.e. lack of discriminant validity) in allowing people to provide responses to all alternatives, irrespectively of whether they apply or not.

A potential limitation of the present study is that it is not based on a random sample. The data consist of a convenience sample collected through an online questionnaire distributed via Facebook which led to some discrepancies compared to the demographic distribution of the Swedish population. An overrepresentation of women amongst the respondents influenced the results, especially for the dog questionnaire. However, although a gender balanced sample would have altered the relative order of attributes, it most likely would not have changed the top ranked attributes. Furthermore, statistics from the US show that women to a larger extent are the primary caregivers for dogs. If this is true also for Sweden it could explain women being overrepresented in the current sample in which a majority of the respondents are caregivers. Another limitation was the low degree of resolution regarding breeds. It is likely that the results would have been different if the answers regarded specific breeds of dogs or cats, since breed differences are pronounced in both species. Furthermore, it is reasonable to assume that the reasons for and expectations of having or wanting to have a cat or a dog would influence the rankings.

## Conclusion

A thorough understanding of how we conceptualize cats and dogs is important as it influences human behavior and well-being as well as how animals are treated, selected and cared for. Although further research is needed in order to fully understand the human conceptualization of cats and dogs, this study highlights the bearers of meanings assigned to cats and dogs, and the relative importance of these. The study thus provides information that can be used in future studies in a range of areas, for example, human health and subjective-wellbeing, human-animal interaction, animal welfare, and the economic value of companion animals.

## Additional file

Additional file 1: Questionnaire. Brief description of the web-based questionnaire and list of questions including response alternatives. (DOCX 15 kb)

## Abbreviations

Attr: attribute
SCP: sum of choice probability
