# Supplementary material for: An empirical examination of the conceptualization of companion animals
Source: BMC Psychol. 2018 May 4;6:15. doi: 10.1186/s40359-018-0228-1 (PMC5934865; doi:10.1186/s40359-018-0228-1)
Supplement: Supplementary file 1 — Questionnaire. Brief description of the web-based questionnaire and list of questions including response alternatives. (DOCX 15 kb) [file 40359_2018_228_MOESM1_ESM.docx]

Data was collected through a web-based questionnaire. Respondents were depending on their answers automatically directed to subsets of questions. Each respondent was asked 13 or 14 questions. The content of the questionnaire is presented below along with the provided response alternatives. The text in italics provides information on which questions different respondents were asked.

**QUESTIONNAIRE:**

**What do you think about dogs and cats?**

This questionnaire is part of an ongoing research project at SLU. You are welcome to participate regardless of whether or not you have dogs or cats. We would appreciate if you want to participate – you can choose to answer questions relating to dogs or cats. The survey consists of a dozen questions and only takes a few minutes to answer. You answer the questions anonymously.

**1. Do you want to answer questions relating to dogs or cats?** Dogs/Cats

*Respondents who answered Cats were automatically directed to question 14 while respondents who answered Dogs continued with question 2.*

**2. When thinking of dogs, what aspects come to mind?**

Describe separately each aspect with one word (or a few words).

**3. Do you have (one or more) dogs in your household?** Yes/No

*Respondents who answered Yes were automatically directed to questions 8-10, 13 and 26-31 while those who answered No were automatically directed to questions 4-7, 11-13, and 26-31.*

**4. Have you been thinking of getting a dog?** Yes/No

*Respondents who answered No were subsequently directed to question 11.*

**5. What are the main reasons you have been thinking of getting a dog?**

Please briefly state each reason separately below.

**6. What dog breed/s have you been thinking of getting?**

If more than one breed please state each breed separately.

**7. Has your household previously had a dog (or dogs)?** Yes/No

*Respondents asked this question were subsequently directed to question 13.*

**8. What were the main reasons that your household decided to get a dog?**

Please briefly state each reason separately below.

**9. What dog breeds do you have in your household?**

If more than one breed please state each breed separately.

**10. How many dogs do you have in your household?** 1 dog/2 dogs/3 dogs/ > 3 dogs

**11. What are the main reasons that you are not interested in getting a dog?**

Please briefly state each reason separately below.

**12. Have you previously had a dog (or dogs) in your household?** Yes/No

**13. Do you have a cat (or cats) in your household?** Yes/No but we have previously had/ No and we have not previously had

**14. When thinking of cats, what aspects come to mind?**

Describe separately each aspect with one word (or a few words).

**15. Do you have (one or more) cats in your household?** Yes/No

*Respondents who answered Yes were automatically directed to question 20-22 and 25-31 while those who answered No were automatically directed to question 16-19, 23-31.*

**16. Have you been thinking of getting a cat?** Yes/No

*Respondents who answered No were subsequently directed to question 23.*

**17. What are the main reasons you have been thinking of getting a cat?**

Please briefly state each reason separately below.

**18. What cat breed/s have you been thinking of getting?**

If more than one breed please state each breed separately.

**19. Has your household previously had a cat (or cats)?** Yes/No

*Respondents asked this question were subsequently directed to question 25.*

**20. What were the main reasons that your household decided to get a cat?**

Please briefly state each reason separately below.

**21. What cat breed/s do you have in your household?**

If more than one breed please state each breed separately.

**22. How many cats do you have in your household?** 1 cat/2 cats/3 cats/ > 3 cats

**23. What are the main reasons that you are not interested in getting a cat?**

Please briefly state each reason separately below.

**24. Have you previously had a cat (or cats) in your household?** Yes/No

**25. Do you have a dog (or dogs) in your household?** Yes/No but we have previously had/ No and we have not previously had

**26. Do you have children in the household?** Yes/No

**27. How many persons does you household consist of in total?**

**28. How old are you?** <20 /20- 29 /30-39 /40-49 /50-65 /66-79 /80 or older

**29. Are you male or female?**  Male/Female

**30. Within which (phone) area code do you live?**

**31. How did you learn about this survey?** ad on Facebook/friend on Facebook/ Facebook of "Framtidens djurhälsa och djurvälfärd"/other way

**The results of the survey will be presented in scientific publications and posted on the web page http://www.slu.se/framtidensdjur**

**Thank you for your participation!**

**Framtidens djurhälsa och djurvälfärd, SLU**
